# Comparative Whole-Genome Analysis of Production Traits and Genetic Structure in Baiyu and Chuanzhong Black Goats

**DOI:** 10.3390/ani14243616

**Published:** 2024-12-15

**Authors:** Jing Luo, Qi Min, Xueliang Sun, Xinyu Guo, Meijun Song, Xuehui Zeng, Jiazhong Guo, Hongping Zhang, Yanguo Han, Li Li

**Affiliations:** 1 College of Animal Science and Technology, Sichuan Agricultural University, Chengdu 611130, China; luojing11032021@126.com (J.L.); 2024102011@stu.sicau.edu.cn (Q.M.); sunxueliang97@163.com (X.S.); guoxinyu1029@163.com (X.G.); 2023102002@stu.sicau.edu.cn (M.S.); z15892314380@163.com (X.Z.); guo@sicau.edu.cn (J.G.); zhp@sicau.edu.cn (H.Z.); 2 College of Animal Science and Technology, Southwest University, Chongqing 400715, China

**Keywords:** goat, production performance, genetic diversity, population structure, selective signal

## Abstract

Natural selection and artificial breeding are crucial methods for developing new animal groups. The Baiyu black goats and Chuanzhong black goats are indigenous goat breeds from distinct ecological regions in Sichuan Province, with dramatically different growth and reproductivity. This study aimed to systematically elucidate the differences in production performance and genetic traits between Baiyu black goats and Chuanzhong black goats. We quantified growth and reproductive attributes for both breeds. Furthermore, we conducted a comprehensive analysis of genetic diversity, population structure, and selection signatures using whole-genome resequencing data. This dataset included 30 individuals from the Baiyu black goat breed, 41 from the Chuanzhong black goat breed, and an additional 59 individuals representing Chengdu grey goats, Tibetan cashmere goats, and Jianchang black goats, totaling 130 individuals across five goat breeds. The comparative analysis of production performance revealed that the weight and body size of Chuanzhong black goats were significantly higher than those of Baiyu black goats (*p* < 0.01). At the same time, the average kidding rate and kid-weaning survival rate of Chuanzhong black goats were also notably superior to those of Baiyu black goats (*p* < 0.01). The Baiyu black goats exhibited a more abundant genetic diversity and distinct genetic differences compared to the Chuanzhong black goat, according to an analysis grounded on genomic variation. The Baiyu black goats are more closely related to Tibetan cashmere goats, whereas Chuanzhong black goats share a closer genetic relationship with Chengdu grey goats. Additionally, we employed the π, Fst, and XP-EHH methodologies to identify genes related to immunity (*TRIM10*, *TRIM15*, *TRIM26*, and *TRIM5*), neurodevelopment (*FOXD4L1*, *PCDHB14*, *PCDHB4*, *PCDHB5*, *PCDHB6*, and *PCDHB7*), reproduction (*BTNL2* and *GABBR1*), body size (*NCAPG*, *IBSP*, and *MKNK1*), and meat quality traits (*SUCLG2* and *PGM5*). These results provide a theoretical basis for further resource conservation and breeding improvement of the Baiyu black goat and Chuanzhong black goat.

## Simple Summary

This study investigates the genetic diversity, population structure, and selection signatures of the Baiyu black goat and Chuanzhong black goat from Sichuan Province, China. These breeds, which are adapted to distinct ecological environments, exhibit significant differences in growth and reproductive performance. We conducted a comprehensive analysis to explore their genetic relationships and performance traits using whole-genome resequencing data from 30 Baiyu black goats and 41 Chuanzhong black goats, along with data from three additional goat breeds. Our results show that Chuanzhong black goats are larger and have higher reproductive success compared to Baiyu black goats. Genetic analysis revealed that the Baiyu black goat possesses greater genetic diversity and is more closely related to Tibetan cashmere goats, while Chuanzhong black goats are genetically closer to Chengdu grey goats. Further, we identified several genes associated with immunity, neurodevelopment, reproduction, body size, and meat quality, which may inform future breeding programs for improved performance and sustainability. This study provides valuable insights for the conservation and genetic improvement of these goat breeds.

## 1. Introduction

Goats (*Capra hircus*) serve as a primary source of meat for humans, and their productive performance is crucial for agricultural development. Furthermore, the genetic resources of goats represent a significant component of biodiversity, which is vital for the sustainable advancement of the livestock industry. The Baiyu black goat is primarily found in Baiyu County of the Ganzi Tibetan Autonomous Prefecture in Sichuan [1]. The Baiyu black goat is a highly adaptable local breed with a long history of domestication by herders and natural selection from wild Tibetan goats. It demonstrates remarkable adaptability to high-altitude climates (altitude ~3500 m) characterized by low oxygen levels, cold temperatures, and intense ultraviolet radiation [1]. The Chuanzhong black goat is another notable local breed in China, predominantly inhabiting the Jintang and Lezhi Counties, which are located in the Chengdu Plain (altitude ~600 m). This breed is celebrated for its large physique, rapid growth rate, robust reproductive capabilities, and exceptional meat production attributes [1]. To adapt to the high-altitude area, extremely harsh climate, and hypoxic environment that the Baiyu black goat lives in, this goat has developed distinct phenotypic traits compared to the Chuanzhong black goat.

Whole-genome sequencing (WGS) technology has been used to evaluate genetic resources and identify important genes in local Chinese goat breeds [2,3,4]. For example, Guo et al. [3] have revealed that Chengdu grey goats, Tibetan cashmere goats, and Chuanzhong black goats (Jintang type) exhibit lower genetic diversity compared to Bezoar ibexes, with selection signatures identifying genes associated with high-altitude adaptation and coat color. Subsequently, Liu et al. [5] found that Xiangdong black goats possess moderate genetic diversity and a relatively large effective population size. In the case of Hainan black goats, studies reveal a high degree of similarity with Leizhou goats, with genes associated with immunity, heat tolerance, meat quality, cashmere production, and stress resistance being identified [6,7]. Collectively, these investigations offer invaluable insights into genetic breeding strategies for goats, which can be harnessed to enhance the productivity of local goat breeds, thereby contributing to agricultural sustainability and economic growth. However, the current assessment of genetic resources for native goat breeds in China is still quite limited, and no reports on whole-genome resequencing studies of the Baiyu black goat have been published.

Considering the distinctive breeding history and environmental adaptability of the Baiyu black goat and Chuanzhong black goat, this study aimed to compare their production traits and characteristics. By analyzing their genetic diversity, population structure, and selection signatures, we provide a deeper understanding of the factors influencing these traits. These findings provide a theoretical foundation for breed conservation, genetic enhancement, and utilization. Additionally, they offer valuable insights for the preservation and breeding of other local goat genetic resources.

## 2. Materials and Methods

### 2.1. Ethics Approval Statement

This work was approved by the Animal Protection and Utilization Committee of Sichuan Agricultural University. Production data measurements and blood sample collection were conducted in accordance with the ‘Experimental Animal Operation Specification’ (Sichuan Agricultural University Document No. 18 [2014]).

### 2.2. Measurement of Production Data and Blood Sample Collection

We measured the production traits of Baiyu black goats (BY, altitude ~3500 m) at the Baiyu Black Goat Conservation Farm in Baiyu County, Ganzi Prefecture, Sichuan Province, and Chuanzhong black goats (JT, altitude ~600 m) at the Chuanzhong Black Goat Breeding Farm in Jintang County, Chengdu, Sichuan Province. We assessed weights at five growth stages: birth, weaning, 6 months old, 1 year old, and adulthood. Adult body size traits, including body height, body length, and chest circumference, were also evaluated. Weight was measured using an electronic scale, with chest girth measured with a soft measuring tape, and body length and height were measured with a measuring stick. Weight was recorded in the morning after a 24 h fast using an electronic scale. The birth weight of kids was recorded immediately after birth. The kidding rate is the number of kids produced by does (female goats), expressed as a percentage of the number of does that gave birth. Additionally, we randomly collected blood samples from 30 Baiyu black goats via the jugular vein, using EDTA-coated tubes for anticoagulation, and transported them back to the laboratory at 4 °C.

### 2.3. Genomic DNA Extraction and Sequencing

Blood samples of the Baiyu black goat were used to extract genomic DNA using the Blood Genome DNA Extraction Kit (Beijing Tiangen Biochemical Technology Co., Ltd., Beijing, China), following the instructions of the manufacturer. The extracted genomic DNA was then assessed for purity and concentration using a UV spectrophotometer. High-quality genomic DNA samples were resequenced at Beijing Novogene Biotechnology Co., Ltd., China. using the Illumina NovaSeq 6000 platform with paired-end sequencing (2 × 150 bp). To characterize the genetic background of the Baiyu black goat, we integrated previously published whole-genome resequencing data (accession number: PRJNA548681 and PRJNA734084) from Chuanzhong black goats (*n* = 41), Tibetan cashmere goats (ZZ, *n* = 14), Jianchang black goats (JC, *n* = 30), and Chengdu grey goats (CM, *n* = 15). Collectively, whole-genome resequencing data from 130 goat individuals were utilized here.

The primary production area (altitude ~1600 m) of the Jianchang black goat is located in Huili City, Sichuan Province. This region shares similar climatic conditions with the production areas of the Baiyu black goat, both of which are situated in the transitional zone between the Yunnan–Kweichow Plateau and the Qinghai–Tibet Plateau. The geographical locations of the Chengdu grey goat and the central production area of the Sichuan black goat are also relatively close. It has been reported that there is genetic flow between the Tibetan cashmere goat and the wild Tibetan goat [8], and the Baiyu black goat is believed to have been domesticated from the wild Tibetan goat.

### 2.4. Mapping and SNP Calling

The raw sequencing data (raw reads) were cleaned using the Trimmomatic (v0.38) software [9] to obtain clean reads. The Burrows–Wheeler Aligner (BWA) version 0.7.17 [10] was used to build a reference genome index file (ARS1, GCA_001704415.1), and the clean reads from the 130 individuals were aligned to the goat reference genome. Duplicate sequences were removed using Picard (v2.21.9) (http://broadinstitute.github.io/picard, accessed on 20 January 2024) and SAMtools (v1.9) [11]. The resulting files were sorted by chromosome coordinates and subjected to local realignment to obtain downstream analysis files in the BAM format. To detect single nucleotide polymorphisms (SNPs), the Genome Analysis Toolkit (GATK) [12] version 4.1.8.1 can be utilized with various modules. The HaplotypeCaller module was utilized for SNP calling, while the GenotypeGVCFs module was employed for genotype refinement. The VariantFiltration module was applied to filter the SNPs. The filtering criteria for SNP variants are as follows: QD < 2.0, QUA < 100.0, SOR > 3.0, FS > 60.0, MQ < 40.0, MQRankSum < −12.5, and ReadPosRankSum < −8.0. After filtering, the resulting high-quality SNPs from all 130 individuals will be annotated for functions using the SnpEff (v5.0) software [13].

### 2.5. Calculation of the Genetic Diversity Index

This study compares the genetic differences between Baiyu black goats and Chuanzhong black goats, as well as other goat breeds, based on whole-genome SNPs. The nucleotide diversity (Pi), runs of homozygosity (ROHs), LD decay, and inbreeding coefficients (Fs) are calculated across five goat breed genomes to assess these differences. Nucleotide diversity was calculated using VCFtools (v0.1.16) [14] with the following parameters: --window-pi 10000 and --site-pi 5000. Linkage disequilibrium analysis was conducted with PopLDdecay (v3.4) using default parameters [15]. The decay plot for linkage disequilibrium was generated using the Plot_OnePop.pl script provided by the PopLDdecay software. Additionally, based on the ROH lengths within the goat genome identified in this study, ROH segments were divided into four categories: 0.1–0.2 Mb, 0.2–0.5 Mb, 0.5–1 Mb, and >1 Mb. To calculate the runs of homozygosity and inbreeding coefficients, the --homozyg and --het commands in PLINK (v1.9) [16] were used, respectively.

### 2.6. Analysis of Population Structure and Phylogenetics

We pruned SNPs exhibiting high levels of pair-wise linkage disequilibrium (LD) using PLINK with the parameter --indep-pair-wise 50 5 0.2, before conducting principal component analysis (PCA) and admixture analysis. PCA was performed using the smartPCA package in EIGENSOFT (v3.0) [17]. ADMIXTURE (v1.3) [18] was used for population structure analysis, with the kinship set from 2 to 4. PLINK was also used to generate the matrix of pairwise genetic distances, which was then employed to construct a neighbor-joining evolutionary tree. The visualization of the evolutionary tree was done using MEGA7 [19] and embellished by iTOL (https://itol.embl.de, accessed on 24 January 2024).

### 2.7. Identification of Selection Signatures

First, we identified selection signatures in Baiyu black goats using nucleotide diversity (π), computed with VCFtools using a window size of 50 kb and a step size of 20 kb. Second, we utilized the fixation index (Fst) and cross-population extended haplotype homozygosity (XP-EHH) methods to detect selection signatures between Baiyu black goats and Chuanzhong black goats. Fst analysis was performed with VCFtools using a window size of 50 kb and a step size of 20 kb. XP-EHH statistics, based on extended haplotypes, were computed using selscan (v1.1) [20]. The LD heatmap for candidate genes was visualized using LDBlockShow [21]. Finally, for the enrichment analysis of candidate genes under selection, we used KOBAS 3.0 (http://bioinfo.org/kobas accessed on 25 January 2024) to conduct the KEGG pathway and Gene Ontology (GO) enrichment analyses.

## 3. Results

### 3.1. Differences in Production Performance Between the Baiyu Black Goat and Chuanzhong Black Goat

**Figure 1:**
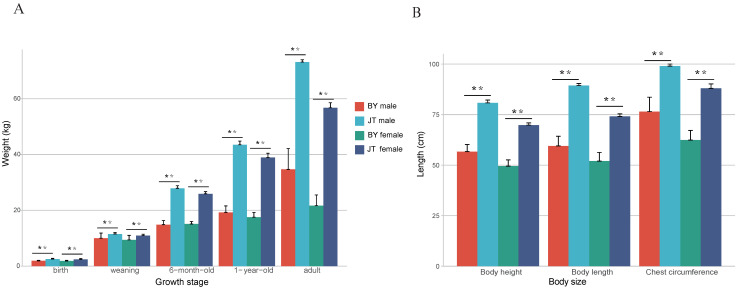
Comparison of main production performance between the Baiyu black goat and Chuanzhong black goat. (**A**) Comparison of weight at different stages between the Baiyu black goat and Chuanzhong black goat. (**B**) Comparison of body size between the Baiyu black goat and Chuanzhong black goat. Note: ** indicates *p*-value less than 0.01.

The primary production performance was assessed through measurements of weight and body size parameters. Among animals of the same age and gender, Chuanzhong black goats consistently exhibited significantly higher weights at all growth stages compared to Baiyu black goats (*p* < 0.01) (Figure 1A, Table S1) and significantly greater height, body length, and chest circumference (*p* < 0.01) (Figure 1B, Table S2). In addition, the average kidding rate was 228% for Chuanzhong black goats and 108% for Baiyu black goats, while the weaning survival rates of their kids were 85.7% and 92.2%, respectively (Table S3). In general, the weaning kids per litter is 1.95 for Chuanzhong black and 1.00 for Baiyu black goats.

### 3.2. Identification and Functional Annotation of SNPs

Utilizing BWA-MEM (version 0.7.13-r1126), we successfully aligned the sequencing reads to the goat reference genome sequence (ARS1.2). An average alignment rate of 99.69% with an average sequencing depth of 8.16× was observed for the 30 Baiyu black goats. In contrast, the average alignment rate for the 41 Chuanzhong black goats was 99.64% with an average sequencing depth of 6.32× (Table S4). By integrating these results with published goat resequencing data, we processed the whole-genome resequencing data of a total of 130 goats from three breeds, resulting in 17,352,739,028 bp of quality-controlled sequencing data. Across all samples, the average alignment rate was 99.40%, and the average sequencing depth amounted to 6.79× (Table S4). These results demonstrate a high degree of sequence homology between the reads and the reference genome, facilitating subsequent genetic and genomic analyses. The functional annotation of SNPs revealed that a substantial proportion of these SNPs were positioned within intergenic regions, accounting for 46.67%, or within intronic regions, comprising 42.89%. Notably, a mere 0.98% of the SNPs were situated within exons (Table S5). This distribution highlights the prevalence of SNPs in non-coding regions of the genome, which may still play significant roles in regulating gene expression and other biological processes [22].

### 3.3. Population Genetic Diversity Assessment

**Figure 2:**
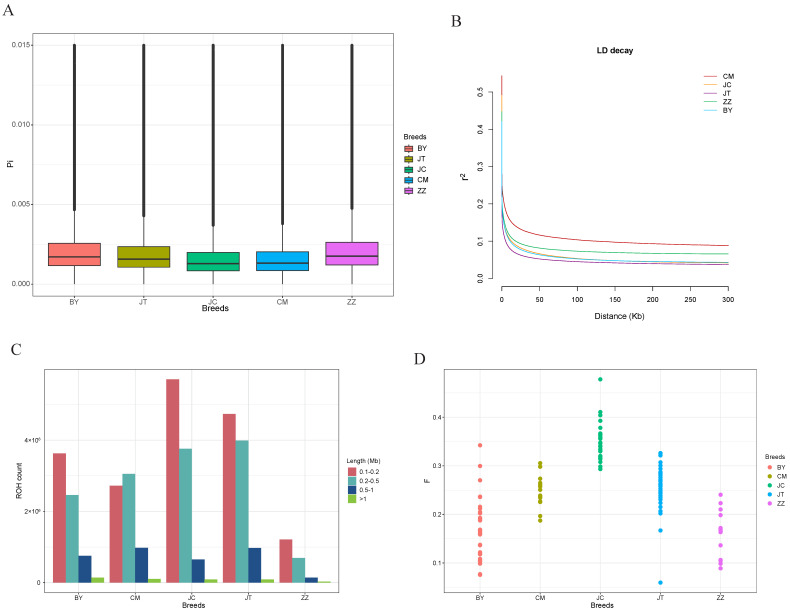
The genome-wide genetic diversity of five goat breeds. (**A**) Box plots of the nucleotide diversity for each breed. (**B**) The average LD decay was estimated from five groups. (**C**) Estimation of the number of ROHs for each group. The ROH length was divided into four categories for statistical purposes: 0.1–0.2 Mb, 0.2–0.5 Mb, 0.5–1 Mb, and >1 Mb, respectively. (**D**) Inbreeding coefficients for each goat breed.

Among these five goat breeds, Baiyu black goats exhibited the highest nucleotide diversity, whereas Jianchang black goats demonstrated the lowest nucleotide diversity (Figure 2A). This indicates that the genetic variation of Baiyu black goats is more abundant. The average genome-wide LD (r^2^) of the Baiyu black goat was higher than that of the Chuanzhong black goat. Chuanzhong black goats exhibited the lowest average LD, while Chengdu grey goats had the highest, followed by Tibetan cashmere goats (Figure 2B). Furthermore, the slower rate of LD decay observed in Baiyu black goats compared to Chuanzhong black goats suggests that Baiyu black goats may have undergone a longer history of domestication or natural selection. Jianchang and Chuanzhong black goats had higher ROH counts compared to Baiyu black goats, indicating greater inbreeding levels. Tibetan cashmere goats exhibited the lowest total count of ROHs. Apart from the Chengdu grey goat, the ROH lengths in other goat breeds predominantly ranged between 0.1 and 0.2 Mb. Chuanzhong black goats have shorter ROHs compared to Baiyu black goats (Figure 2C), indicating a higher level of recent inbreeding in Chuanzhong black goats, which was confirmed by the inbreeding coefficient based on genome heterozygosity (Figure 2D) [23]. These results collectively indicate that Baiyu black goats exhibit a higher level of genetic diversity compared to Chuanzhong black goats.

### 3.4. Population Structure Characterization and Relationships

**Figure 3:**
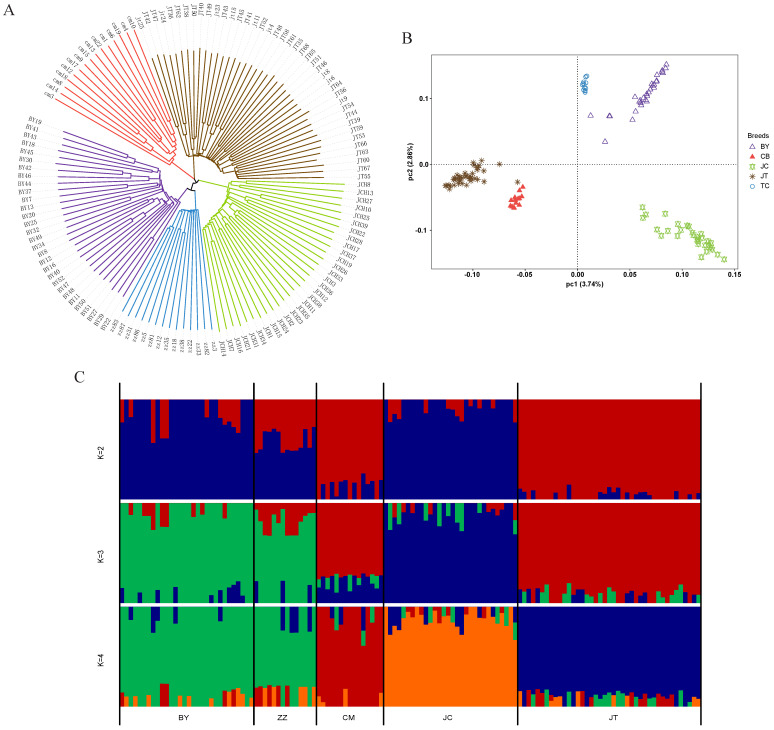
Population genetic and phylogenetic relationship comparisons of five goat breeds. (**A**) Neighbor-joining tree among 130 samples from five breeds. (**B**) Principal component analysis comprising 130 individuals across five distinct goat breeds. (**C**) Genetic structure of five breeds using ADMIXTURE, when K ranged from 2 to 4.

To elucidate the phylogenetic relationships among Baiyu black goats, Chuanzhong black goats, and their respective affinities with other goat breeds from Sichuan and Tibet, we employed genomic SNPs to construct a neighbor-joining (NJ) tree, perform principal component analysis (PCA), and conduct admixture analysis (Figure 3). The NJ tree results revealed the closest kinship between Baiyu black goats and Tibetan cashmere goats, while Chuanzhong black goats exhibited the strongest affinity with Chengdu grey goats (Figure 3A). This finding supports the hypothesis that Baiyu black goats are descended from domesticated wild Tibetan goats. The PCA results were consistent with the phylogenetic tree (Figure 3B). In the admixture analysis, when K was set to 2 or 3, Baiyu black goats and Tibetan cashmere goats exhibited identical ancestral compositions, while Chuanzhong black goats and Chengdu grey goats share a common ancestral makeup. This suggests that both Baiyu black goats and Tibetan cashmere goats evolved from the same ancestral lineage. However, at K = 4, Chuanzhong black goats and Chengdu grey goats were genetically distinguished from each other (Figure 3C). These results highlight the significant genetic differentiation between Baiyu and Chuanzhong black goats, implying the potential utility of the identified genetic markers for the development of selective breeding strategies aimed at improving targeted traits in both breeds.

### 3.5. Genome-Wide Selective Sweep Signature

**Figure 4:**
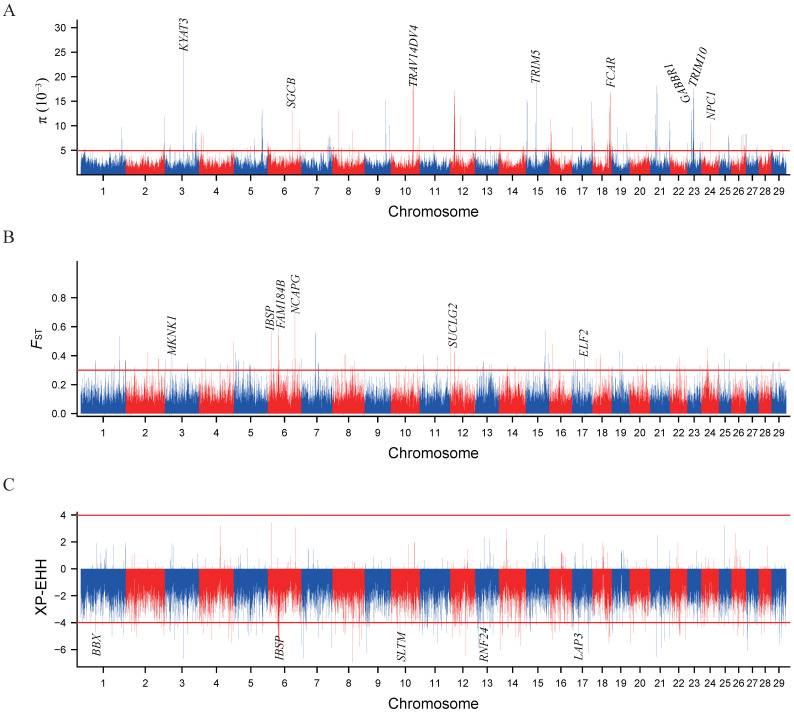
The signature selection in the Baiyu black goat and Chuanzhong black goat. (**A**) Manhattan plot of selective sweeps by π in the Baiyu black goat. (**B**) Manhattan plot of selective sweeps by Fst in the Baiyu black goat and Chuanzhong black goat. (**C**) Manhattan plot of selective sweeps by XP-EHH in the Baiyu black goat and Chuanzhong black goat.

We applied nucleotide diversity analysis (π) to detect genomic regions related to selection in Baiyu black goats (Figure 4A and Table S6). With a selection threshold set at 0.005, a total of 23 candidate genes were identified in Baiyu black goats. These genes encompass those associated with immunity (*TRIM10*, *TRIM15*, *TRIM26*, and *TRIM5*) [24,25] and metabolism (*KYAT3*) [26,27], which may contribute to the ability of Baiyu black goats to adapt to high-altitude environments, as well as genes related to reproduction (*BTNL2* and *GABBR1*) [28,29] and body weight (*DCHS1* and *NPC1*) [30,31].

**Figure 5:**
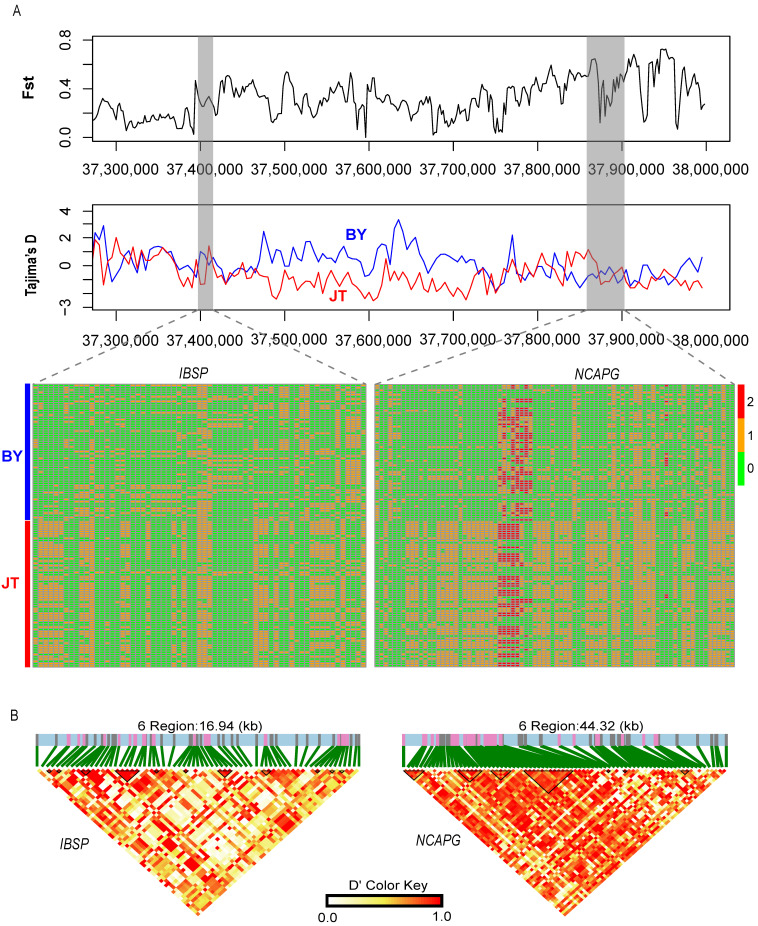
The signature selection in the Baiyu black goat and Chuanzhong black goat. (**A**) Plots of Fst and Tajima’s D, as well as haplotype diversity, for the IBSP and NCAPG genes. (**B**) The LD heatmap of the IBSP and NCAPG gene regions.

**Figure 6:**
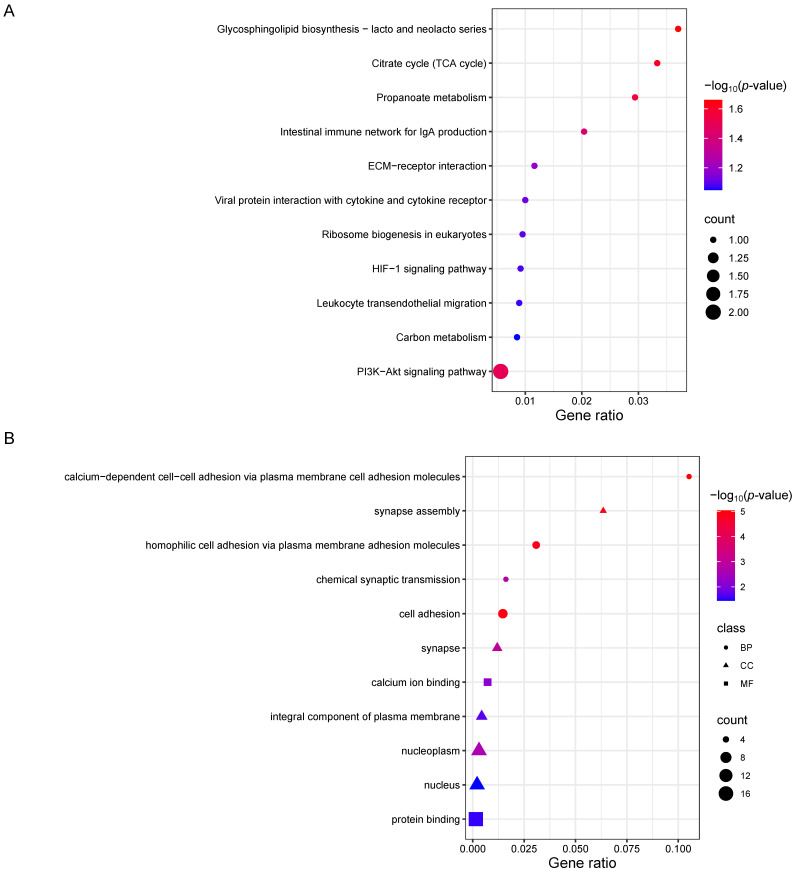
The KEGG and GO enrichment analyses of 41 overlapped genes. (**A**) The KEGG enrichment analysis of 41 overlapped genes. (**B**) The GO term analysis of 41 overlapped genes.

The Fst and XP-EHH methodologies were further employed to detect signatures of selection between Baiyu black goats and Chuanzhong black goats (Figure 4B,C). We obtained 231 and 182 putatively selected genes from the Fst and XP-EHH methods, respectively (Tables S7 and S8). An overlap of the genes screened by both methods yielded 41 strongly selected genes. Among these, genes associated with body size (*NCAPG*, *IBSP*, and *MKNK1*) [32], neural development (*FOXD4L1*, *PCDHB14*, *PCDHB4*, *PCDHB5*, *PCDHB6*, and *PCDHB7*) [33,34], and meat quality (*SUCLG2* and *PGM5*), were found to be under intense selection. To verify the reliability of candidate genes, we examined the variations in the *IBSP* and *NCAPG* gene regions. The results show a discrepant haplotype diversity pattern and Tajima’s D between the Baiyu black goat and Chuanzhong black goat (Figure 5). We performed functional enrichment analysis using KEGG pathways and Gene Ontology (GO) for the overlapped genes (Figure 6). The KEGG enrichment analysis revealed significant enrichment of 41 selected genes in pathways such as glycosphingolipid biosynthesis—lacto and neolacto series, the citrate cycle (TCA cycle), propanoate metabolism, the PI3K-Akt signaling pathway, and the intestinal immune network for IgA production (*p* < 0.05) (Table S9 and Figure 6A). Notably, the GO enrichment analysis highlighted multiple functional categories and pathways (corrected *p*-value < 0.01), spanning cellular components, biological processes, and molecular functions. The cellular components include synapse assembly, synapse, and nucleoplasm (Table S10). The biological processes involve calcium-dependent cell–cell adhesion via plasma membrane cell adhesion molecules, cell adhesion, homophilic cell adhesion via plasma membrane adhesion molecules, and chemical synaptic transmission. In terms of molecular functions, calcium ion binding stands out (Figure 6B). These enriched GO terms further underscore the potential roles of these genes in neural development and function.

## 4. Discussion

Superior production performance can meet market demands and improve economic efficiency, while substantial genetic diversity contributes to biological evolution, species adaptation, and resource conservation. These two factors collectively drive the sustainable development of the livestock industry and preserve biodiversity. Our analysis of the productive and reproductive performance data of Chuanzhong black goats and Baiyu black goats reveals that Chuanzhong black goats exhibit significantly higher weight, body measurements, average kidding rate, and kid-weaning survival rates compared to Baiyu black goats. This finding is consistent with the research outcomes reported by Qisha et al. [1], underscoring the superior production and reproductive performance of the Chuanzhong black goat.

The high nucleotide diversity in Baiyu black goats highlights their potential as a valuable genetic resource for future breeding and conservation efforts. Liu et al. [35] conducted a genetic diversity analysis using AFLP markers on eight goat breeds sourced from Chengdu, Lezhi, Jintang, Baiyu, Yingshan, Hejiang, Jiangan, and Jialing in Sichuan, China. Their findings concurred that the Baiyu black goat possessed the highest gene diversity index (0.0888), indicating substantial genetic diversity within this breed. Furthermore, the LD decay in Baiyu black goats was relatively gradual compared to that of Chuanzhong black goats, which may be attributed to the smaller effective population size resulting from less favorable environmental conditions. Notably, Baiyu black goats exhibit fewer total ROHs, short ROHs, and a lower inbreeding coefficient compared to Chuanzhong black goats. These observations suggest that Baiyu black goats may have effectively mitigated inbreeding in their breeding management or have undergone less selective pressure [36]. Lower levels of inbreeding and selective pressure are generally associated with higher genetic diversity, which in turn confers greater potential for adaptation to varying environmental conditions and enhanced resilience to diseases [37]. A comprehensive evaluation of these genetic diversity indices underscores that Baiyu black goats possess higher genetic diversity than Chuanzhong black goats. After prolonged selective breeding and intensive breeding, the Chuanzhong black goat has achieved a relatively high level of production performance. In contrast, the Baiyu black goat, which has undergone less intensive artificial selection and has an incomplete breeding system, exhibits higher genetic diversity compared to the Chuanzhong black goat. This diversity has preserved a more varied genetic reservoir within the Baiyu black goat population. With directed selection, the Baiyu black goat holds the potential to be bred into varieties that are more resistant to diseases, better adapted to climatic variations, and better suited to meet the dynamic demands of the market.

Baiyu black goats are closely related to Tibetan cashmere goats, whereas the Chuanzhong black goats (Jintang type) are more closely related to Chengdu grey goats. Previous analyses utilizing AFLP, RAPD, and microsatellite markers consistently indicated a close genetic proximity between the Chengdu grey goat and Chuanzhong black goat, while Baiyu black goats were genetically distant from both. Additionally, based on maternally inherited mtDNA D-loop sequences, it was also revealed that the Chuanzhong black goat and Baiyu black goat belong to distinct genetic groups, characterized by a notable genetic distance between them [38,39]. These findings align with the results of the present study and indicate a distant phylogenetic relationship between the Baiyu and Chuanzhong black goats, likely due to their differing breeding histories, geographical locations, and environmental niches.

Altitude differences can lead to varying environmental stressors such as temperature, climate, hypoxia, and UV radiation, which in turn can influence the genetic makeup of populations [8]. We conducted a selection sweep analysis on Baiyu black goats utilizing nucleotide diversity (π) as a metric, which led to the identification of genes associated with immunity (*TRIM10*, *TRIM15*, *TRIM26*, and *TRIM5*), reproduction (*BTNL2* and *GABBR1*), weight (*DCHS1* and *NPC1*), and metabolism (*KYAT3*). It has been reported that the *TRIM* gene family potentially underlies the resilience to African swine fever by modulating innate antiviral responses [40]; It is plausible that these genes contribute to the ability of goats to withstand infectious diseases, thereby enhancing their overall fitness and survival in their native environment. *GABBR1*, a neurotransmitter receptor, has been implicated in schizophrenia [41]. In addition, a genome-wide association study reveals that *GABBR1* is a key gene for the number of offspring born alive in the pink-eyed white mink [29]. In the context of Baiyu black goats, *GABBR1* may play a role in reproductive processes, potentially influencing fertility and litter size, which are critical for the persistence of the breed. The protein encoded by the *NPC1* gene plays a pivotal role in cholesterol transportation and lipid metabolism [31]. In cattle, the *NPC1* gene may also be implicated in growth development and reproductive processes [42,43]. Moreover, *NPC1* was correlated with yearling weight, participating in controlling the steady state of energy metabolism in Chinese fine-wool sheep [31]. In Baiyu black goats, *NPC1* may similarly influence the growth rate, feed efficiency, and reproductive performance, contributing to their adaptability to various environmental conditions. The *KYAT3* gene has undergone intense selection in Baiyu black goats, with reports suggesting its role in sheep metabolism making them more adaptable to adverse environmental conditions [26]. Collectively, we have identified several candidate genes associated with immunity and energy metabolism, which are likely to be significant in the adaptation of Baiyu black goats to high-altitude environments.

The Fst and XP-EHH analyses identified genes associated with body size (*NCAPG*, *IBSP*, and *MKNK1*) and meat quality (*SUCLG2* and *PGM5*), suggesting strong selective pressures on traits critical for economic and production purposes. Notably, *NCAPG* and *LCORL*, frequently found adjacent to each other across multiple animal genomes, form the NCAPG–LCORL locus, recognized as a genetic determinant of adult height. Selective sweeps and genome-wide association studies have consistently linked NCAPG–LCORL to body size (weight/height) in species such as cattle [32], horses [44], pigs [45], dogs [46], and goats [47]. The *IBSP* gene has been implicated in traits including ear size [48], carcass traits [49], and bone weight in cattle [50]. It has also been linked to growth, milk production, and carcass traits in sheep [51]. In goats, *IBSP* is associated with milk production and hair follicle/hair growth [47,52]. *MKNK1*, involved in cellular growth and protein synthesis [53], has been correlated with average daily gain in pigs [54]. The differential expression of these genes is likely to underlie the variation in body size between Baiyu and Chuanzhong black goats. *SUCLG2*, which is associated with pork meat quality [55], displays differential expression in both yak muscles (with varying degrees of tenderness) but also in the biceps femoris and longissimus dorsi muscles of goats [56,57]. *PGM5* plays a crucial role in myofibril formation, maintenance, and remodeling [58]. The combined influence of these two genes may be implicated in contributing to the differences in meat quality observed between the Baiyu black goats and the Chuanzhong black goats.

## 5. Conclusions

This study conducted a comprehensive comparative analysis by leveraging production data and whole-genome resequencing data from Baiyu black goats and Chuanzhong black goats. Significantly, Chuanzhong black goats exhibited superior production traits, whereas Baiyu black goats demonstrated a higher level of genomic genetic diversity. Interestingly, the genetic backgrounds of Baiyu black goats and Chuanzhong black goats are markedly distinct, with Baiyu black goats showing closer genetic proximity to Tibetan cashmere goats than to other black goat breeds. Furthermore, we identified candidate genes associated with body size, meat quality, immunity, and neural development. These findings provide a theoretical foundation for the conservation, genetic improvement, and exploitation of both Baiyu black goats and Chuanzhong black goats as valuable genetic resources.

## Data Availability

The study-generated datasets are available in the Genome Sequence Archive (https://ngdc.cncb.ac.cn/gsa/ accessed on 10 January 2024). The Bioproject accession number is PRJCA028669.

## Supplementary Materials

The following supporting information can be downloaded at https://www.mdpi.com/article/10.3390/ani14243616/s1: Table S1: Comparison of body weight at different stages between the Baiyu black goat and Chuanzhong black goat; Table S2: Comparison of body size between the Baiyu black goat and Chuanzhong black goat; Table S3: Comparison of main reproductive performance indicators between the Baiyu black goat and Chuanzhong black goat; Table S4: Sequencing data statistics of five goat breeds; Table S5: Functional annotation of the identified SNPs in all breeds; Table S6: A summary of genes from π in Baiyu black goats; Table S7: A summary of genes from Fst in Baiyu black goats and Chuanzhong black goats; Table S8: A summary of genes from XP-EHH in Baiyu black goats and Chuanzhong black goats; Table S9: KEGG pathway of 41 genes overlapping Fst and XP-EHH; Table S10: The GO enrichment of 41 genes overlapping Fst and XP-EHH.
